# McKillip Veterinary College

**Published:** 1901-04

**Authors:** 


### McKILLIP VETERINARY COLLEGE.

THE fifth annual commencement was held in the College Auditorium, 1639 Wabash Avenue, Chicago, at 2 P.M., March 29, 1901. The exercises were opened with prayer by Rev. Johnson Meyers, which was followed by an address to the graduating class by Prof. E. Merillat. The class programme consisted of a salutatory address by H. B. Treman; "Class Prophecy," by B. O. Minge; "Class History," by H. F. Emich, and "Valedictory," by R. D. Scurfield. The Secretary, L. A. Merillat, announced the standing of members of the senior class, which was as follows: Twenty-one members were entitled to graduation. R. D. Scurfield was awarded the Lovejoy prize for having the best grade for three years' work; William Schumacher, second; H. H. Cohenour, third; T. P. Brankin, honors. C. D. McGilvray received the first prize for best senior grade; J. F. Olweiler, second; R. D. Scurfield, third, and honors to William Schumacher, N. Clark, H. B. Treman, H. H. Cohenour. President M. H. McKillip conferred the degree of M.D.V. upon the following members of the senior class: R. D. Scurfield, Manitou, Manitoba; H. F. Emich, Lima Grove, Ind.; F. W. Buecher, Chicago, Ill.; H. H. Cohenour, Pana, Ill.; W. H. Perrigo, Danville, Ill.; W. I. Gass, Sheboygan, Wis.; J. Gallagher, Chicago, Ill.; T. P. Brankin, Joliet, Ill.; H. B. Treman, Storm Lake, Iowa; B. O. Minge, Faunsdale, Ala.; R. L. Kann, Mechanicsburg, Pa.; Oscar Hartnagle, Victoria, B. C.; C. D. McGilvray, Binscarth, Man.; T. A. Kragness, San Francisco, Cal.; N. Clark, Valparaiso, Ind.; F. C. Willitt, Morristown, N. J.; J. M. Simpson, Mombaccus, N. Y.; J. R. Batch, Chicago, Ill.; J. F. Olweiler, Elizabethtown, Pa.; William Schumacher, Lagrange, Ill.; T. Lambreichts, Montevideo, Minn.

This closes one of the most prosperous and successful terms in the history of McKillip Veterinary College. The number of students in attendance during the present school year was seventy-seven.

